# Continuous Driver’s Gaze Zone Estimation Using RGB-D Camera

**DOI:** 10.3390/s19061287

**Published:** 2019-03-14

**Authors:** Yafei Wang, Guoliang Yuan, Zetian Mi, Jinjia Peng, Xueyan Ding, Zheng Liang, Xianping Fu

**Affiliations:** 1 Information Science and Technology College, Dalian Maritime University, Dalian 116026, China; wangyafei@mail.dlut.edu.cn (Y.W.); yuan@dlmu.edu.cn (G.Y.); mizetian@dlmu.edu.cn (Z.M.); jinjiapeng@dlmu.edu.cn (J.P.); dingxueyan_meow@dlmu.edu.cn (X.D.); zliang@dlmu.edu.cn (Z.L.); 2 School of Microelectronics, Dalian University of Technology, Dalian 116024, China

**Keywords:** RGB-D camera, ICP, head pose, gaze estimation

## Abstract

The driver gaze zone is an indicator of a driver’s attention and plays an important role in the driver’s activity monitoring. Due to the bad initialization of point-cloud transformation, gaze zone systems using RGB-D cameras and ICP (Iterative Closet Points) algorithm do not work well under long-time head motion. In this work, a solution for a continuous driver gaze zone estimation system in real-world driving situations is proposed, combining multi-zone ICP-based head pose tracking and appearance-based gaze estimation. To initiate and update the coarse transformation of ICP, a particle filter with auxiliary sampling is employed for head state tracking, which accelerates the iterative convergence of ICP. Multiple templates for different gaze zone are applied to balance the templates revision of ICP under large head movement. For the RGB information, an appearance-based gaze estimation method with two-stage neighbor selection is utilized, which treats the gaze prediction as the combination of neighbor query (in head pose and eye image feature space) and linear regression (between eye image feature space and gaze angle space). The experimental results show that the proposed method outperforms the baseline methods on gaze estimation, and can provide a stable head pose tracking for driver behavior analysis in real-world driving scenarios.

## 1. Introduction

Driver distraction and inattention are the key factors that cause traffic accidents. Distracted driving increases the probability of crashes as the drivers shift their attention from driving. To recognize and prevent these types of potential dangers, driving behavior monitoring plays an increasingly significant role in Advanced Driver Assistance Systems (ADAS), and high level ADAS can provide higher forms of automation, in which drivers are even expected to glance away from the primary operational task and be guided to get through some critical situation.

**Figure 1:**
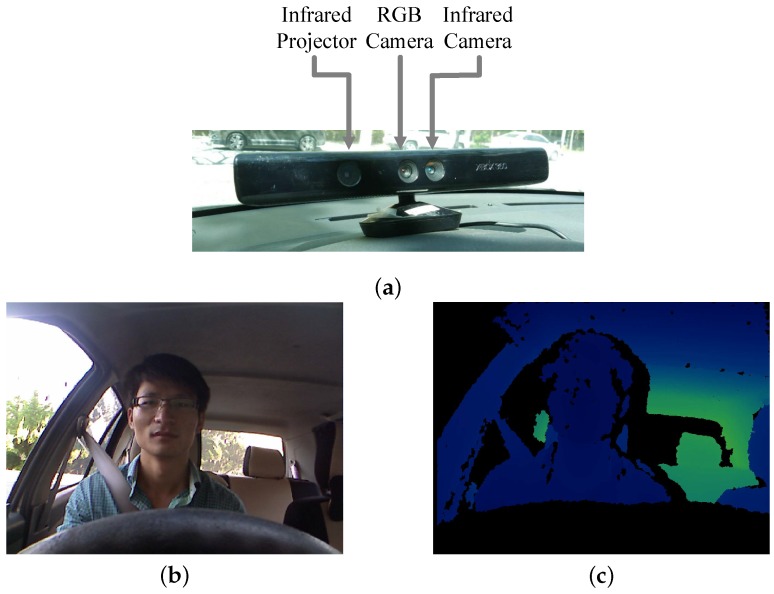
(**a**) Exterior appearance of RGB-D camera (Kinect v1) [4], and corresponding RGB-D data (**b**,**c**) obtained by it.

Human-centric driving monitor technologies can be divided into two categories, intrusive-sensing technologies and remote-sensing technologies. While the intrusive-sensing technologies [1] detect head motion from attached head orientation sensors, some biomedical sensing technologies [2,3] measure the signals from the driver immediately and intuitively, but disturb the driver in the process, leading to inconvenience complaints. Vision-based applications usually mount the remote cameras inside the vehicle, and are capable of monitoring the driver in a non-contact and non-invasive way. These applications benefit from the advance in information technologies, and can present computer vision algorithms based on low-cost sensors. Figure 1 shows the typical RGB-D camera and the corresponding RGB-D data.

In the driver context, the dynamics of a driver’s head and eye are potential to present where or what he/she is looking at. The allocation of a driver’s gaze is linked to a driver’s current attention. Therefore, studying a driver’s gaze direction and fixation has been extensively applied for visual distraction detection and understanding driver activities, and in natural driving, many drivers move both their heads and eyes when looking at the target. Many gaze tracking systems have been proposed for monitoring driver’s attention state [5]. Detailed surveys of gaze estimation and head pose estimation can be seen in Refs. [6,7].

Coarse gaze direction based on a driver’s head orientation is usually acceptable in vision-based driver behavior monitoring systems. The probability of driver gaze is often generated by a gaze zone estimator. The discrete gaze zones are defined as the in-vehicle components where drivers are looking at, such as windshields, rear-view mirrors, side mirrors, etc. Since head pose contributes to gaze direction, many gaze zone estimation methods consider head orientation as the indicator of the gaze zone in a convenient manner, and parts of many studies treat the gaze zone estimation as a combination of head pose estimation (head pose value) and gaze estimation (gaze angle value of eyeball) in three degree of freedom (Euler angle), yaw, pitch and roll. This is consistent with real driving behavior in natural driving, resulting in many drivers moving both their heads and eyes when they are looking at the target.

From the perspective of sensor information, driver gaze zone estimation systems fall into one of two categories: systems using RGB/Grayscale cameras, and RGB-D cameras.

RGB or Grayscale Cameras: Most systems that use RGB or Grayscale cameras are largely relying on precise localization of facial features. Constrained Local Model (CLM) is one of the Facial Landmark Detection (FLD) methods, and has been commonly employed to extract and analyze the head pose and its dynamic in single [8] or multiple camera systems [9,10]. The driver’s face is detected in an unpredicted environment and further location of the frontal facial landmark points under model constraints (typical instances are various feature points annotated around face contour, eyes, eyebrows, nose and mouth). In order to provide robust representation against illumination and accelerate the detection speed, Vicente et al. [11] expressed face shape by Supervised Descent Method (SDM) using an SIFT descriptor and analyzed the geometric configurations of facial landmark points to estimate the head pose. After FLD process, head pose vector or facial feature landmarks are extracted as training features for gaze zone estimation.

Besides, FLD uses eye alignment to locate the eye region for eye pose estimation. By assuming the human eyeball as a spherical 3D eye model with a constant radius, there are only several parameters needed. One of important parameter is pupil center. As the pupil is darker than other parts of the eye region, Fridman et al. [12] used an adaptive threshold of the histogram of the eye image to segment the pupil blob, but it does not work well in the non-uniform varying lighting conditions. On the low resolution eye image, Trawari et al. [10] detected the iris center (same as the eyeball center) using the HoG descriptor. This method trained the local patches of the eye image under different light, but to a great extent, needed some image processing steps to ensure its detection quality. Vicente et al. [11] used SDM tracker to detect eye landmarks including six eye contour points and the pupil. The eye estimation followed a 3D eye model-based approach in their work.

However, it is still critical for the above systems to obtain depth information, especially when solving the 3D head pose from the 2D images depending on the detected landmarks and their relative 3D configurations with a weak perspective projection model. To address the varying changes of head position and head rotation, Ultrasonic sensors [13,14] or dual cameras [15] are used as extra devices for generating more information to compensate for head movement.

RGB-D Cameras: Standard RGB cameras can take advantage of color information, but lack depth information due to inherent hardware restrictions. The great challenges for such works are the illumination vulnerability under poor environmental conditions where light and shade bring negative effects. To overcome some of these difficulties, RGB-D cameras are applied to obtain both RGB images and more information using point-cloud-based sensors. RGB-D cameras can synchronously capture RGB images and depth images. Different RGB-D cameras are implemented by stereo cameras, structured light, time-of-flight or laser scanners. The more expensive the sensors are, the more accurate point-cloud they achieve. RGB-D cameras benefit from the depth appearance or point-clouds that generated by themselves to build gaze zone estimation systems.

To handle the point-clouds, ICP (Iterative Closet Points) algorithm [16,17] that is used for iterative registration between the free-form three-dimensional rigid point-cloud surfaces, has been applied to calculate the rotation matrix and offset vector between source face template and target face templates. Peláez C. et al. [18] presented a gaze zone estimation system to estimate head pose by analyzing the projection of three-dimensional point-cloud based on ICP. With continuous iterative correction, ICP can minimize the distance from the source point-cloud to the target point-cloud within a given three-dimensional space. However, when the point-cloud level grows larger, the time cost increases dramatically.

Therefore, Bär et al. [19] used Newton method to optimize the ICP solution process. A Newton method is favorable for a faster convergence than a gradient descent method. Multi-templates were used in point-cloud alignment to compute the head pose, subsequently, driver’s gaze angle was analyzed on the eye gaze model. Experimental results show that their system can obtain robust estimation of head pose than single-template system, but the Newton method requires more strict initial value, thus, their system suffers from the problem of falling into a local solution. More studies show that in the process of ICP alignment, adding a filter (such as temporal filter [20], Kalman Filter [21], etc.) to track and learn its state at the next timeframe can solve ICP anisotropic conversion more effectively and stably. Particle Swarm Optimization (PSO) algorithm [22] can solve this through the cooperative behavior of a generation of evolutionary particles. Although PSO has achieved better results, its response is too slow.

Based on depth image appearances, the training regression model for head pose estimation can be constructed by labelling a large number of training sample. Fanelli et al. [23,24] built a random forest regression model and tested depth image appearances with different scanning accuracy. Random forest regression was used to map the depth images to the continuous head pose space by probabilistic voting, in which random sampling samples were adopted to avoid over-fitting. Their results are sensitive to the depth image acquisition and preprocessing, and poor solutions will result in the case of online testing. Breitenstein et al. [25] used the depth appearance of the nose region to predict the head pose, collected reference appearance during the offline stage, and then calculated the errors between the candidate depth and the current input. However, these methods have not been applied in driver gaze zone estimation systems. One of the most important reasons is that the depth appearances maybe incomplete in real driving environments, due to the illumination changes and occlusions.

For gaze estimation or head pose estimation using RGB-D cameras [26,27,28], RGB and depth images can also be used in different processing events. Usually, a depth image is used for foreground segmentation, head localization and object tracking, while the RGB image is used for eye localization and feature extraction. For example, Cazzato et al. [29] located the facial landmark points and position of pupil center in RGB images, and predicted head pose by ICP alignment. The human line of gaze was estimated by oriented feature points surrounding the eye. Mora et al. [30,31] also provided gaze system combining of head pose estimation and gaze estimation, but they used appearance-based gaze estimation methods instead of model-based methods. However, these methods only have better estimation accuracy in the case of a frontal face; the errors increase on low-resolution eye images under free movement.

This work focuses on the applicable gaze zone estimation system with RGB-D cameras performance in a real-world driving environment, and adapts for variants of ICP to align a driver’s face templates. The highlights of the paper are shown below:

- An application-oriented ICP-based point-clouds alignment solution for continuous driver gaze zone estimation using RGB-D camera is proposed, applying multi-zone templates for target face templates revision, and particle filter tracking with auxiliary sampling for initializing and updating the best transformation of source face template; at the same time, the head state is tracked and learned to cope with high rotation velocities under natural head turns, providing reliable head pose value in both yaw, pitch and roll.
- A novel appearance-based eye gaze estimation with two-stage neighbor selection is utilized, avoiding the inaccurate pupil center localization in a real remote driving environment and the vulnerable eye gaze model under very large head rotation. The proposed eye gaze estimation method treats gaze prediction as a combination of cascaded nearest neighbor query and local feature regression.

A summary of driver gaze zone estimation using an RGB-D camera is provided in Table 1. Compared with the previous gaze zone detection systems using RGB-D cameras, the proposed system presents continuous resolution not only for the gaze zone estimation, but also for the head pose estimation and gaze estimation. Unlike the multi-template ICP in Ref. [19], they ensured the transformation of the point-clouds by averaging the results of multiple templates. However, the target templates will be changed due to the varying illumination changes and large head rotations and presence of partial occlusion of eye glasses or light source. We revise multi-zone ICP for balancing the templates’ revision in the real driving scenario. Furthermore, particle filter tracking is used for initialize and update the best transformation of ICP. Unlike model-based gaze estimation methods, which have disadvantages due to the vulnerability under large head movement, the appearance-based gaze estimation method is a better alternative. Furthermore, we conduct the gaze estimation as a two-stage nearest neighbor selection from both head pose space and image feature space. This structure makes it more efficient. The proposed system outputs the final gaze zone index by classifying the gaze angle with head pose compensation.

The rest of this paper is organized as follows. Section 2 introduces our driver gaze zone estimation system that combines the head pose tracking and gaze estimation. The details of implementing multi-zone ICP-based head pose estimation appear in Section 2.1. Section 2.2 presents head state tracking by auxiliary particle filter. Section 2.3 shows the proposed appearance-based gaze estimation with neighbor selection. In Section 3, the proposed system is evaluated and some practical issues regarding the implementation are considered. Finally, Section 4 gives a brief conclusion.

## 2. Proposed System

**Figure 2:**
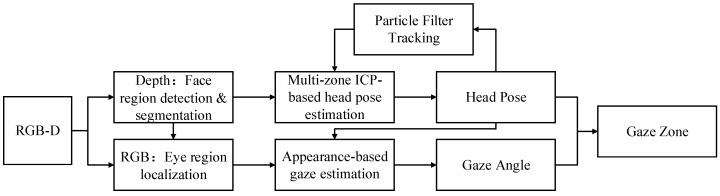
Overview of the proposed system.

This paper presents a combination of multi-zone ICP-based head pose tracking, and appearance-based gaze estimation to build a continuous driver gaze zone detection system (as shown in Figure 2). These two parts have been handled in Depth image and RGB image, respectively.

**Figure 3:**
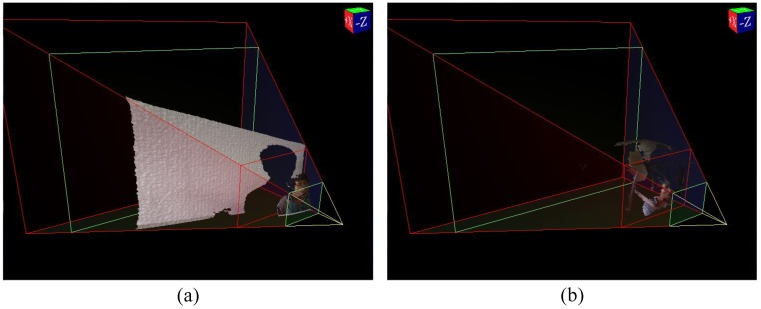
Point-cloud at different distance: (**a**) in-door, (**b**) in-vehicle.

On the depth image, the scene depth information can be easily obtained. Therefore, as shown in Figure 3, the face region in the foreground is segmented from the driving environment with adaptive minimum distance restrictions. Simultaneously, face detection using Viola–Jones method [32] is used to judge whether a driver’s face has been searched and further shrink the face region. At this point, the three-dimensional point-cloud data of face templates has been extracted more precisely and can basically meet the needs of subsequent operations. Some pre-processing is applied to remove outliers, reduce noises, and preserve the geometric characteristics of point-cloud at the same time. After smooth filtering on the depth image, its corresponding three-dimensional point-cloud is generated for rigid transformation. This point-cloud is called the source template.

To estimate head pose under large head rotation, a multi-zone ICP-based method is proposed. By taking advantage of the least squares techniques, source point-cloud and corresponding reference point-cloud templates are aligned under iterative operation, alignment, comparing, adjusting, re-alignment, re-comparing, and re-adjusting. Proper templates at different gaze zones can reduce the templates accumulative error under large head motion. In order to solve the problem that the iteration result does not converge, the head state is tracked and learned by auxiliary particle filtering. The ICP-based point-cloud alignment is then initialized by the prediction value of head state. Head pose in Euler angle will output by the recent head transformation. It should be noted that the reference templates for a multi-zone can be captured when a driver sits down and glances at the labeled center of the pre-defined self-centered gaze zone.

On the RGB image, an eye region is localized in the face region. Due to the scale of a driver’s face region not changing dramatically, the eye region is easier to be captured in the constraint of face detection. The normalized eye images have been mapped into the image feature space, while head pose that is generated in the head poses estimation have been mapped into the head pose space. Appearance-based gaze estimation using neighbor selection is utilized, in which both head pose and eye image features contribute to gaze prediction. By two-stage nearest neighbor searching in both head pose and image feature space, more relevant image features can be found for building the mapping relationship between image feature space and gaze angle space. Final gaze direction is obtained as the gaze angle with head pose compensation. Then, gaze zone estimation is a classification of final gaze direction by *k*-Nearest Neighbor.

Detailed information about head pose estimation, head state tracking and gaze estimation is described in the following chapters.

### 2.1. Multi-Zone ICP-Based Head Pose Estimation

The human face region is considered as rigid surface of three-dimensional model without deformation. Regardless of the perspective transformation and scale factor, only takes into account the linear transformation and translation transformation of the coordinate system, the rigid transformation between two human face point-cloud set data is defined as:

(1) $$\begin{array}{c} T=\left[\begin{array}{cc} R & t\\ 0 & 1 \end{array}\right]\; \end{array}$$

where, T is a 4×4 matrix, R is a 3×3 rotation matrix, t is a 3 × 1 translation vector. The rotation matrix of cloud point alignment is a continuous right multiplication process of three orthogonal rotation matrix with a determinant of 1.

(2) $$\begin{array}{c} R=R_{x}R_{y}R_{z},\;t={\left[\begin{array}{ccc} t_{x} & t_{y} & t_{z} \end{array}\right]}^{T} \end{array}$$

(3) $$\begin{array}{c} R_{x}=\left[\begin{array}{ccc} 1 & 0 & 0\\ 0 & \cos\alpha & \sin\alpha \\ 0 & -\sin\alpha & \cos\alpha \end{array}\right]\;R_{y}=\left[\begin{array}{ccc} \cos\beta & 0 & \sin\beta \\ 0 & 1 & 0\\ -\sin\beta & 0 & \cos\beta \end{array}\right]\;R_{z}=\left[\begin{array}{ccc} \cos\gamma & \sin\gamma & 0\\ -\sin\gamma & \cos\gamma & 0\\ 0 & 0 & 1 \end{array}\right]\; \end{array}$$

To solve the transformation matrix T, ICP algorithm is applied for aligning different point-clouds [33]. During data acquisition and rigid transformation, unavoidable data noise always exists, and causes the alignment of target point-cloud and source point-cloud not to achieve accurate results. Therefore, in order to improve the accuracy of calculation, it is necessary to find as many effective corresponding point pairs as possible, to constrain the transformation matrix.

The main steps of the basic ICP algorithm for point-cloud alignment are: (1) search the nearest neighbor point pairs between two point-cloud using the correspondence estimation; (2) calculate the transformation matrix by the least squares method in an iterative way with all the valid point pairs, until it meets the convergence conditions.

#### 2.1.1. Nearest Neighbor Search

**Figure 4:**
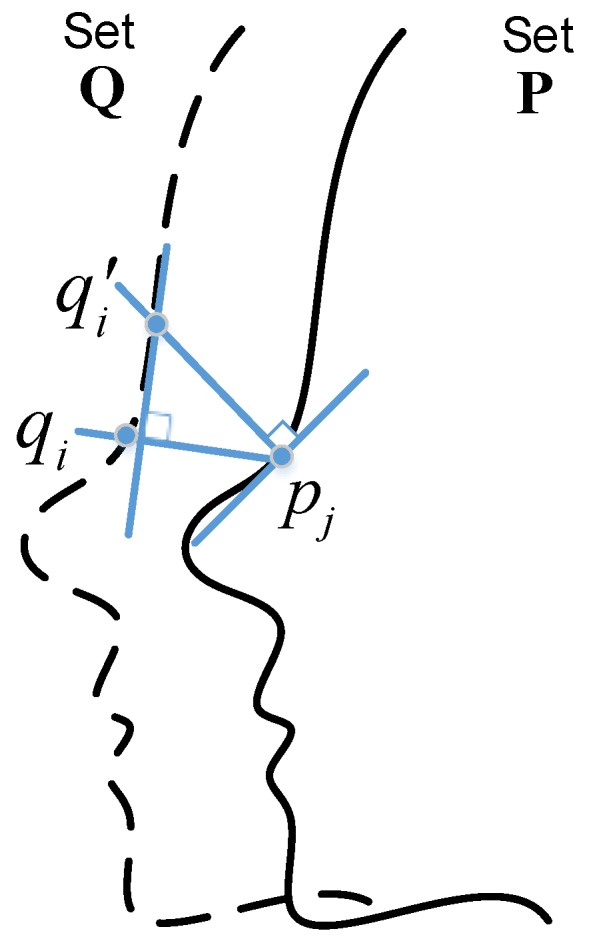
Point-to-Plane nearest neighbor search for point-cloud alignment.

In a given point-cloud set P and Q, a set of nearest neighbor point pairs $\left(q_{i},p_{j}\right)$ can be extracted, where $q_{i}\in Q$ and $p_{j}\in P$. Thus, $\forall q_{i}\in Q$, at least one closest point $p_{j}\in P$ exists. In order to reduce the computational complexity of the rapid search, the corresponding point pairs are computed by the normal under the minimum distance constraints, and the obtained nearest neighbor at this time is an approximated nearest point, rather than the ground-truth nearest point. Figure 4 shows a schematic diagram of nearest neighbor search process based on the Point-to-Plane method [34]. Firstly, based on the normal of reference point $p_{j}$ at the point-cloud P, the intersection $q_{i}^{\prime }$ of the normal at the point-cloud Q can be found. Then, make the tangent plane of $q_{i}^{\prime }$, and draw the vertical line between the point $p_{j}$ and the tangent plane. Finally, compute the intersection point $q_{i}$ at the point-cloud Q. Thus far, a point pair $\left(q_{i},p_{j}\right)$ is extracted.

Through the Point-to-Plane nearest neighbor search, the found neighbor point pairs are not strictly constrained one by one correspondence. That means the different points on the source point-cloud maybe have built a pair relationship with the same point on the reference point could. Moreover, because of the influence of data noise, partial outliers are produced, and confuse the related point pairs. Furthermore, to eliminate the interference of outliers and build a stable point pairs relationship, the reciprocal correspondence point pairs are selected after the filtering method smooth the noise in the space. The reciprocal correspondence point pairs are intersection of two sets of nearest neighbor points pairs, exchange the reference point-cloud source and the reference point-cloud source.

In summary, reciprocal correspondence nearest neighbor point pairs search strategy is utilized in the proposed point-cloud alignment method, which accelerates the search speed and reduces index complexity, generating effective point-cloud pairs for further transformation computation of point-cloud alignment.

#### 2.1.2. Iterative Computing of Transformation

The calculation process of transform matrix is as follows. Firstly, the space mapping reconstruction error function is defined by least square method using the generated nearest neighbor point pairs. Then, a coarse transformation matrix is optimized and solved by minimizing the error function. By projecting the source point-cloud to the coordinate system of the reference point-cloud, the new source point-cloud for next repeat is gotten. Each repeat process is a combination of the optimization of transformation matrix and nearest neighbor point pairs searches for the new source point-cloud. The fine transform matrix will be gotten until it satisfies the convergence condition.

**Figure 5:**
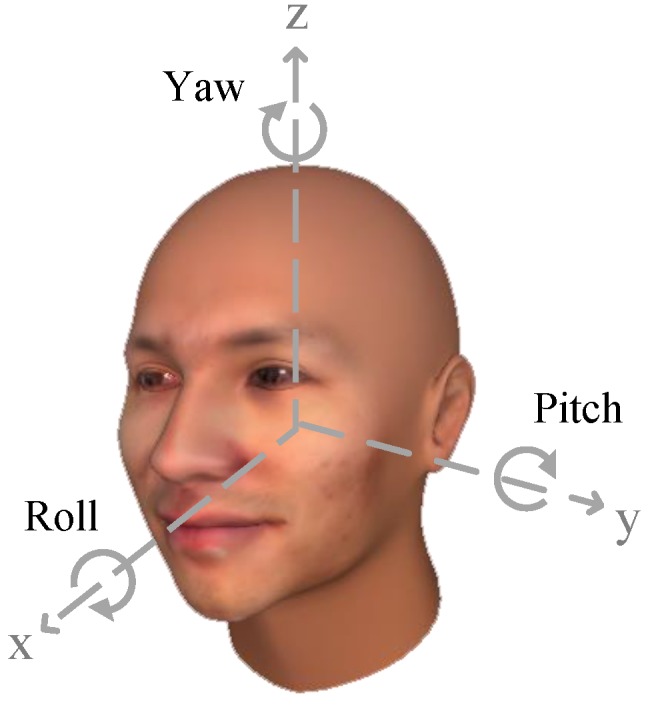
Three degrees of freedom of head pose.

When the final transformation matrix is solved, the rotation angle of head pose in Euclidean space can be calculated using the right-hand Cartesian coordinate system (as shown in Figure 5).

(4) $$\begin{array}{c} \alpha =\arctan\left(\frac{R_{32}}{R_{33}}\right),\;\beta =\arctan\left(\frac{-R_{31}}{\sqrt[{}]{R_{32}^{2}+R_{33}^{2}}}\right),\;\gamma =\arctan\left(\frac{R_{21}}{R_{11}}\right)\; \end{array}$$

where, $R_{ij}$ denotes the element of R at *i* row *j* column. α, β and γ denotes the yaw, pitch and roll of driver’s head pose, respectively.

In general, there are large rotations of a driver’s head in the real driving condition, but the vast majority of the related head poses are concentrated on several gaze regions, such as the left mirror, right mirror, rear-view mirror, windshield, etc. All these areas are known as the gaze zone.

To reduce the cumulative error of ICP iteration, a multi-zone ICP-based head pose estimation method is proposed by applying templates of different gaze zones in continuous tracking. To accelerate the iterative process of ICP, particle filtering is used in tracking the head pose, initializing the coarse transformation matrix. Detailed descriptions of particle filtering are in Section 2.2. All reference templates are collected with ground-truth head pose values and represent different gaze zones. The head pose estimation system first initializes the reference template with zero angle in head pose Euclidean space, then calculates the Euclidean distance of the estimated head pose and the corresponding head pose of the reference templates, and determines the current template index by choosing the 1-Nearest Neighbor. Typically, a driver’s head pose will vary depending on the driving behavior.

The steps of the proposed head pose estimation method are shown in the Algorithm 1.

**Algorithm 1:** Multi-zone ICP-based Driver’s Head Pose Estimation.
1:Initialize multiple cloud point templates for different driver gaze zone$P=\{P_{1},P_{2},\cdot \cdot \cdot ,P_{m}\}$.2:For each new cloud point Q, calculate the predicted head state by Particle Filter tracking, and get the initial value of (R,t): $\left(\hat{R},\hat{t}\right)$.3:Update the coarse head pose value (α,β,γ) based on Equation (4) with $\left(\hat{R},\hat{t}\right)$.4:Update the current gaze zone index *m* of templates using *k*-NN method.5:Search the nearest point pairs between Q and $P_{m}$ using Nearest Neighbor Search algorithm:
$$\begin{array}{c} \forall p_{j}\in P_{m},\;\exists q_{i}=\underset{q_{i}^{\prime }\in Q}{\arg\;\min}\mathrm{nnsp}\left(p_{j},q_{i}^{\prime }\right) \end{array}$$
where, nnsp(·) is a Point-to-Plane Nearest Neighbor Search function with correspondence strategy.  6:Calculate the optimal transformation $\left(R_{best},t_{best}\right)$ via minimize the reconstruction error between Q and $P_{m}$ by:
$$\begin{array}{c} \left(R_{best},t_{best}\right)=\underset{\hat{R},\hat{t}}{\arg\;\min}e\left(\hat{R},\hat{t}\right)=\underset{\hat{R},\hat{t}}{\arg\;\min}\sum_{N_{P}}∥\hat{R}P_{m}+\hat{t}-Q∥ \end{array}$$
$\left(R_{best},t_{best}\right)$ is computed in a iterative process, until the reconstruction error is below the given threshold τ.7:According to the Right-hand Cartesian Coordinate system, update the fine head pose value (α,β,γ) based on Equation (4) with $\left(R_{best},t_{best}\right)$.8:Tracking the head state by particle filter and goto Step 2


### 2.2. Head State Tracking by Particle Filter

**Figure 6:**
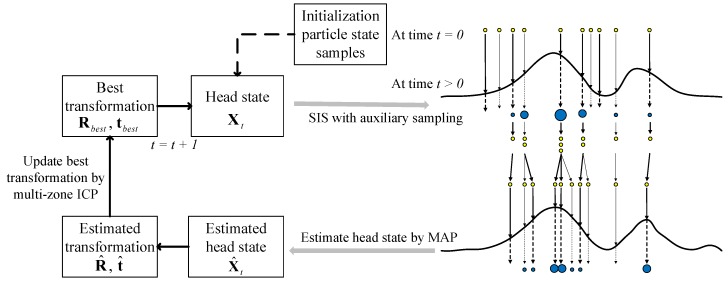
Head state tracking by particle filter.

Particle filter is a nonlinear filtering algorithm based on Bayesian estimation, and has unique advantages in dealing with parameter estimation and state tracking. In this chapter, it is assumed that the driver’s face is a rigid mesh, and we treat the alignment of the 3D point-cloud between source and templates as motion variant of head pose state. Therefore, the driver’s head state dynamic model is established based on particle filter, and the translation and rotation of a head in a given state space is tracked and learned by particle filters. In order to solve the particle impoverish and weight assignation problem of particle filter, an auxiliary sampling method is used in Sequential Importance Sampling (SIS). Figure 6 shows the overall framework of head state tracking by particle filter.

#### 2.2.1. State Space Model

In state space, an unobservable driver’s head state is part of time series dynamics, and defined as $X_{1:t}=\left\{X_{1},X_{2},\cdot \cdot \cdot ,X_{t}\right\}$. At the same time, some observations $Y_{1:t}=\left\{Y_{1},Y_{2},\cdot \cdot \cdot ,Y_{t}\right\}$ are made at continuous time points; it is assumed that all the state sequence is a Markov chain. In this case, similar to [35], the driver state space model can represent the process of the time series, the main composition of which is:

(5) $$\begin{array}{c} X_{t}=F_{t}\left(X_{t-1},U_{t}\right)↔\overset{\mathrm{Transition}\mathrm{Density}}{\overset{︷}{f_{t}\left(X_{t}|X_{t-1}\right)}}\\ Y_{t}=G_{t}\left(X_{t},V_{t}\right)↔\overset{\mathrm{Observation}\mathrm{Density}}{\overset{︷}{g_{t}\left(Y_{t}|X_{t}\right)}} \end{array}$$

where, $X_{t}=\left(A_{t},v_{t}\right)$ is the driver’s head state. $v_{t}$ is a two-dimensional vector consisting of line velocity and angular velocity. The driver’s head state $X_{t}$ and data $Y_{t}$ are assumed to be generated by nonlinear functions $F_{t}$ and $G_{t}$, respectively, of the state and noise disturbances $U_{t}$ and $V_{t}$, and $A_{t}=\left(t_{x},t_{y},t_{z},\alpha ,\beta ,\gamma \right)$ is a six-dimensional vector, which is consisting of head displacements of the axis $t_{x}$, $t_{y}$, $t_{z}$, and head rotation α, β, γ. Based on Equation (4), $A_{t}$ can be convert into ICP initial value R and t of the rigid transformation.

Generally, the driver’s typical head motions can be divided into two parts. One is static state that focuses on the straight ahead direction without offset. The other motion is the linear dynamics that moves from one position to another. These situations can be modeled as mixed driver’s head state [36]: $X_{t}^{*}$$=(X_{t},\tau_{t})$, where $X_{t}=\left(1-\tau_{t}\right)X_{t}^{\left(1\right)}+\tau_{t}X_{t}^{\left(2\right)}$, and $\tau_{t}$ is a binary sign of velocity, with a value of 0 or 1. $X_{t}^{\left(1\right)}=\left(\begin{array}{cc} 1 & 0\\ 0 & 0 \end{array}\right)X_{t-1}^{\left(1\right)}+\left(\begin{array}{c} u_{t}^{\left(1\right)}\\ 0 \end{array}\right)$ denotes the state with a speed of almost zero, while $X_{t}^{\left(2\right)}=\left(\begin{array}{cc} 1 & 1\\ 0 & 1 \end{array}\right)X_{t-1}^{\left(2\right)}+\left(\begin{array}{c} u_{t}^{\left(2\right)}\\ 0 \end{array}\right)$ denotes the state of constant velocity. $u_{t}^{\left(1\right)}$ and $u_{t}^{\left(2\right)}$ are random variables that account for changes of the head state from different i.i.d. stochastic sequences.

The driver’s head state observation model is defined as $Y_{t}=T_{\in }X_{t}+V_{t}$, where $T_{\in }$ is conversion matrix between two space, and $V_{t}$ is the noise at time *t*. The distribution of $\tau_{t}$ is based on the rotational speed of the driver head. When $\tau_{t}=1$, $d\left(Y_{t},Y_{t-1}\right)>\varepsilon_{1},d\left(A_{t},A_{t-1}\right)>\varepsilon_{2}$, where $\varepsilon_{1}$, $\varepsilon_{2}$ are the threshold of rotation speed, it means that the movement of head exceeds the range. Otherwise, when $\tau_{t}=0$, the head is stay still.

#### 2.2.2. Particle Filter Tracking

On the basis of the above driver’s head state space model, auxiliary particle filter method is applied to improve the probability distribution of the driver’s head state at the new time point. Relying on the probability inference of posterior probability density, the joint probability density of driver’s head state and observed state is given as:

(6) $$\begin{array}{c} p_{0:T,0:T}\left(X_{0:T},Y_{0:T}\right)=p_{0}\left(X_{0}\right)g\left(Y_{0}|X_{0}\right)\times \prod_{t=1}^{T}f\left(X_{t}|X_{t-1}\right)g\left(Y_{t}|X_{t}\right) \end{array}$$

where, $p_{0}$ is the initial probability density of $X_{0}$. According to the driver’s head state conversion model and observation model, the states and the data are from randomly sampling process. Their sample pathes $\left({\tilde{x}}_{0},{\tilde{x}}_{1},\cdots ,{\tilde{x}}_{T}\right)$ take the initial value ${\tilde{x}}_{0}∼p_{0}\left(X_{0}\right)$, and otherwise ${\tilde{x}}_{t}∼f_{t}\left(x_{t}|X_{t}={\tilde{x}}_{t-1}\right)$. The corresponding ${\tilde{y}}_{0}∼g\left(Y_{0}|X_{0}\right)$ with a initial value ${\tilde{y}}_{0}∼g\left(Y_{0}|X_{0}\right)$, and otherwise $\tilde{y_{t}}∼g_{t}\left(y_{t}|X_{t}={\tilde{x}}_{t}\right)$.

Since it is not possible to accurately obtain the current driver’s head state distribution trend p(X), the standardized distribution of importance q(X) is utilized as an alternative, and the weight of current state sample data is updated by the previous observed driver’s head state. For the *i*-th sample weight $w_{t}^{\left(i\right)}$, $w_{t}^{\left(i\right)}=\frac{p_{t}\left(x_{t}^{\left(1\right)}\right)}{q_{t}\left(x_{t}^{\left(i\right)}\right)}$. By $p\left(X_{t}=x_{t}|Y_{1:t}=y_{1:t}\right)=\frac{p\left(y_{t}|x_{t}\right)p\left(x_{t}|y_{1:t-1}\right)}{p(y_{t}|y_{1:t-1})}$, and set $\sigma_{t|0:t-1}=\int_{{ℜ}^{N_{x}}}g_{t}\left(Y_{t}=y_{t}\right)p_{t|0:t-1}\left(X_{t}=x|y_{0:t-1}\right)dx$, therefore the joint probability density can be computed by

(7) $$\begin{array}{c} p_{0:t|0:t}\left(X_{0:t}=x_{0:t}|Y_{0:t}=y_{0:t}\right)=\frac{g_{t}\left(x_{t}|Y_{t}\right)p_{0:t|0:t-1}\left(x_{0:t}|y_{0:t}\right)}{\sigma_{t|0:t-1}\left(y_{t}|y_{t-1}\right)} \end{array}$$

Since it is impossible to sample according to the density function $p_{0:t|0:t}\left(X_{0:t}|Y_{0:t}\right)$, the *N* samples ${\tilde{x}}_{0:t}^{\left(i\right)},i=1,2,\cdots ,N$ are selected based on the probability density $q_{0:t|0:t}\left(X_{0:t}|Y_{0:t}\right)$, and the sampling importance weights are computed by

(8) $$\begin{array}{c} {\tilde{w}}_{t}^{\left(i\right)}=\frac{p_{0:t|0:t}\left({\tilde{x}}_{0:t}|y_{0:t}\right)}{q_{0:t}\left(x_{0:t}|y_{0:t}\right)} \end{array}$$

All these weights are standardized and mapped to the interval [0, 1].

The weights will gradually probably fail after a long time of running, so the importance re-sampling is added after each weight calculation. In order to facilitate the survival of particles in the next moment, auxiliary sampling is used in the standard re-sampling process of the probability distribution of the driver’s head state. It is assumed that the joint posterior probability function at the time point can be well approximated using the Dirac measure of that time point.

A rough approximation function $f\left(dx_{t}|x_{t-1}^{\left(i\right)}\right)\approx \delta_{\mu_{t}^{\left(i\right)}}\left(dx_{t}\right)$ is used in the re-sampling, then the joint probability density can be approximated by

(9) $$\begin{array}{c} p_{0:t|0:t}\left(dx_{0:t-1}|y_{0:t}\right)\approx \sum_{i=1}^{N}g\left(y_{t}|\mu_{t}^{\left(i\right)}\right)w_{t-1}^{\left(i\right)}\delta_{x_{0:t-1}^{\left(i\right)}}\left(d_{x_{0:t-1}}\right) \end{array}$$

At this point, the generalized importance ratio of particles is given as

(10) $$\begin{array}{c} {\tilde{w}}_{t}^{\left(i\right)}=\frac{{\tilde{w}}_{t-1}^{\left(i\right)}}{{\tilde{v}}_{t-1}^{\left(i\right)}}\times \frac{g\left(y_{t}|x_{t}^{\left(i\right)}\right)f\left(x_{t}^{\left(i\right)}|x_{t-1}^{\left(i\right)}\right)}{q_{t}\left(x_{t}^{\left(i\right)}|x_{t-1}^{\left(i\right)},y_{t}\right)} \end{array}$$

Compared with the standard sequential importance sampling, the sampling in this chapter revises the important weights by $\frac{1}{v_{t-1}^{\left(i\right)}}$, and the weight ratios by $\frac{w_{t-1}^{\left(i\right)}}{v_{t-1}^{\left(i\right)}}$. In this way, during the re-sampling process before sampling, the particles predicted at the previous moment are extended to increase particle diversity at the current moment and to reduce the variance of the importance weights, producing a more accurate estimate.

At this point, the driver’s head state transition density $f_{t}\left(X_{t}|X_{t-1}\right)$ can be estimated based on the observation density $g_{t}\left(Y_{t}|X_{t}\right)\propto \frac{1}{B}exp\left(-\lambda d\left(Y_{t},T_{\in }X_{t}+V_{t}\right)\right)$, where *B* is a standardized constant. Therefore, the current driver’s head state $X_{t}$ is computed by the weighted average of the samples $\hat{X_{t}}=\sum_{n=1}^{N}w_{t}^{\left(n\right)}{\tilde{x}}_{t}^{\left(n\right)}$.

### 2.3. Appearance-Based Gaze Estimation Using Neighbor Selection

The proposed appearance-based gaze estimation is modeled in a local neighbor-based regression way, which contains three steps: feature extraction, two-stage neighbor selection and PLSR for gaze regression. The facial landmark detection and eye region localization contribute in extracting the eye images and head pose for gaze prediction. Neighbor selection seeks the neighbor of test sample in a training dataset. The nearest neighbors have similar properties in head pose and image feature. Gaze regression based on PLSR (Partial Least Squares Regression) is then employed to model using these neighbor samples.

The driver’s face always appears fully in the field of view. After the face region, which takes the bounding box, is localized, it is easy to obtain the eye region according to the landmarks, and head angle values are computed through trigonometry operations using elements from a rotation matrix. The head vector is converted from the rotation matrix to its axis-magnitude representation by Rodrigues Transform, which can also be used to transform three basic vectors to a rotation matrix.

The success of neighbor selection is highly depent on the appropriate construction of neighbor feature space. However, finding the proper neighbors from large scale eye image dataset is still a challenging problem. Because eye appearance is sensitive to head movement, head pose feature is significant for appearance-based gaze estimation with free head movement. Similar gaze direction under the same head pose for the same subject has a closet pupil center.

Here, gaze directions are regressed under similar head pose and image feature using the local manifold.

**Figure 7:**
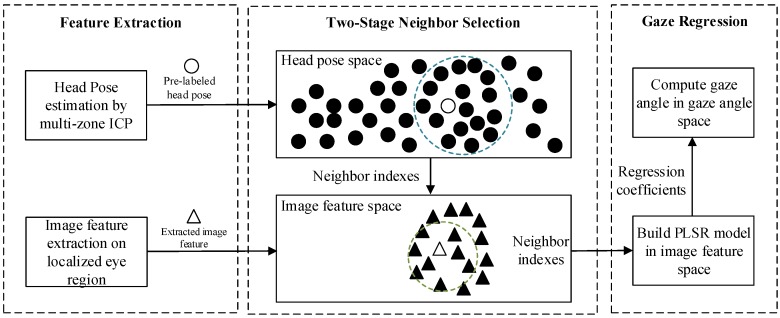
Appearance-based gaze estimation using neighbor selection.

As shown in Figure 7, the proposed neighbor selection method consists of a double *k*-NN query in different feature spaces. This work provides a simple version of our previous work [37]. Here, Raw features are used as the appearance descriptor. A training dataset with query table has been built, in which each item of table contains index, eye image and its corresponding features (head pose and image feature). Image features with less nearest neighbors are found in the scope of the test data. The found image features are used as neighbor samples appearance for gaze local regression.

Previous local regression method based on *k*-NN usually estimates gaze angle using the mean of selected neighbors, which ignores the correlation between samples and gaze angles. To handle this, PLSR is utilized to reduce the dimensionality and project the gaze angle data onto components of maximum covariance with the image feature data. It is a combination of two methods: partial least squares (PLS) analysis and multiple linear regression. Furthermore, the statistically inspired modification of the PLS method (SIMPLS) algorithm is used in the gaze local regression for its competitiveness on large scale dataset [38].

Given eye appearances $X_{feature}\in R^{k\times n}$ and gaze directions $Y_{gaze}\in R^{3\times n}$, then the gaze regression can be modeled as [39] by

(11) $$X_{feature}=T_{feature}P_{feature}^{T}+E$$

(12) $$Y_{gaze}=U_{gaze}G_{gaze}^{T}+F$$

where, $T_{feature}$ and $U_{gaze}$ are the scores of X and Y, respectively. $P_{feature}$ and $G_{gaze}$ are the loadings of $X_{feature}$ and $Y_{gaze}$, respectively. E and F are the residual matrixs.

PLS matrices $T_{feature}$ and $U_{gaze}$ contain latent variables which are calculated as the linear combination of $X_{feature}$ and $Y_{gaze}$. Assume $T_{feature}=X_{feature}W$ and $U_{gaze}=Y_{gaze}N$. Thus, according to Ref. [39], PLSR model is reformulated as follows:

(13) $$Y_{gaze}=X_{feature}B+F^{★}$$

where, $B=W{\left(P_{feature}^{T}W\right)}^{-1}N$. The covariance between score vectors is maximized in each iteration of PLS, the *i*-th components of W and N can be computed by

(14) $$\begin{array}{c} w^{\left(i\right)},n^{\left(i\right)}=\arg\;\max_{w,n}n^{T}\left(Y_{gaze,0}^{T}X_{feature,0}\right)w\\ s.t.\;|w|=1,|n|=1,t_{feature}^{\left(i\right)}⊥t_{feature}^{\left(j\right)}\;\forall j<i \end{array}$$

where, $t_{feature}^{\left(i\right)}$ is *i*-th score vector of $X_{feature}$. $Y_{gaze,0}$ and $X_{feature,0}$ are the refined value of $Y_{gaze}$ and $X_{feature}$, that have subtracted the mean vector of themselves. When the regression coefficients B is obtained, the predicted gaze angle can be determined by $Y_{test}=X_{test}B$, where $X_{test}$ is the image feature of test sample.

## 3. Experimental Results and Discussion

This section evaluates the accuracy of the proposed system in different tasks on natural driving data. First, we report the performance of our head pose estimation in different gaze zones. Second, we compare our gaze estimation with head pose compensation to other baseline methods. Finally, we evaluate the driver’s gaze point and analyze the driver’s attention transfer probability between different gaze zones.

### 3.1. Experiment Setup and Data Sources

Evaluation is performed on a collection of video sequences of driving subjects with depth measurements. All image data (RGB images and Depth images) are collected from natural and on-road driving using the Kinect v1. For the Kinect’s installation in the real driving environment, it needs to be placed at the position that neither interfere the driver’s operation, nor occlude the effective field of view of the depth sensor, and the Kinect is designed for indoor applications with a range of detection at 0.8–4.0 m and with a field of view at $57^{\circ }\left(Horizontal\right)\times 43^{\circ }\left(Vertical\right)$.

**Figure 8:**
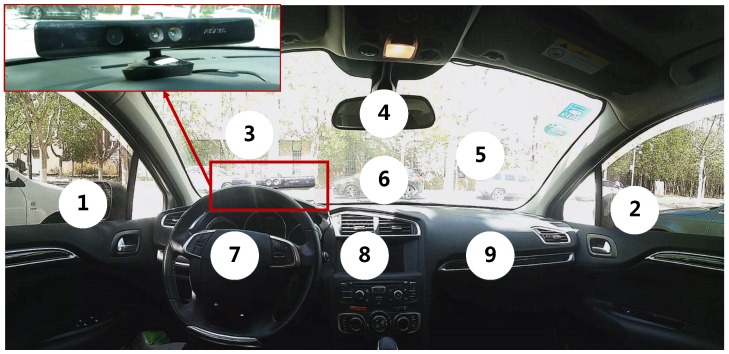
Driver gaze zone partition in real driving environment.

Therefore, in the evaluation, the Kinect is mounted facing the driver with the placement above the instrument board and in front of the windshield (as shown in Figure 8). It captures both depth and RGB video stream of the face view at 30 frames/s. The resolution of the Depth image is 320×240, while the RGB image resolution is 640×480. After image interpolation, they are of the same resolution. Afterwards, all images in which the face images are out of camera range or the eyes blink are discarded automatically. In this manner, the image dataset delivers almost 50,000 RGB data and Depth data from single driver in natural driving. For each frame, a depth image, a color image, the ground truth head pose, and gaze zone index are provided.

To provide the precise ground truth head pose value, the IMU (Inertial Motion Units), consists of three MPU6050 sensors, and is attached on the driver’s head to track its respective motion. IMU sensor outputs continuous head rotation angle by interior gyroscope with Kalman filter at 50 frames/s. To balance the sampling frequency of the IMU and the Kinect, IMU data is sub-sampled after data acquisition. The Kinect and the IMU are connected to a laptop via the USB interface and serial port, respectively. The whole system is powered by a 220-V portable power bank, and runs on one laptop with 2.30 GHz Intel Core i5 CPU and 8 GB RAM.

To evaluate the gaze direction, particular regions of interest are annotated as gaze zones in our dataset. All gaze zones are partitioned in the front of driver seat and contain most normal driving behavior movements. The considered gaze zones are left mirror, right mirror, rear-view mirror, instrument board, steering wheel, navigation system, glove box and several regions of the windshield, as shown in Figure 8.

**Figure 9:**
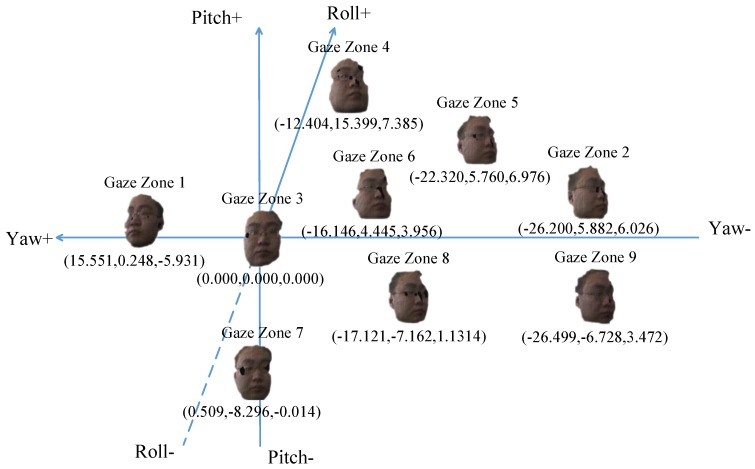
Point-cloud templates of different gaze zone.

Head pose estimation needs to be initialized under a condition in which the zero point of yaw, pitch and roll represents straight-ahead head direction as gaze zone 3. Due to each driver having his or her distinct head behavior when he/she turns face to the fixed gaze zone, we calibrate the head pose in one time when the driver looks the windshield region at a specific direction. There are some error degrees about −0.7 to +0.6 in yaw, −0.3 to +0.3 in pitch and −0.4 to +0.3 in roll. This initialization is performed using the first 100 frames, and the center point is determined by a weighted average of estimated head pose. Typically, the personalized head pose values for each gaze zone are stabilized. The nine calibrated head poses and their corresponding gaze zone are shown in Figure 9. The gaze zone index is displayed above the templates, and the head pose angle in Euclidean angle space is shown under the template in Euler angle (yaw, pitch, roll).

### 3.2. Results of Head Pose Estimation

**Figure 10:**
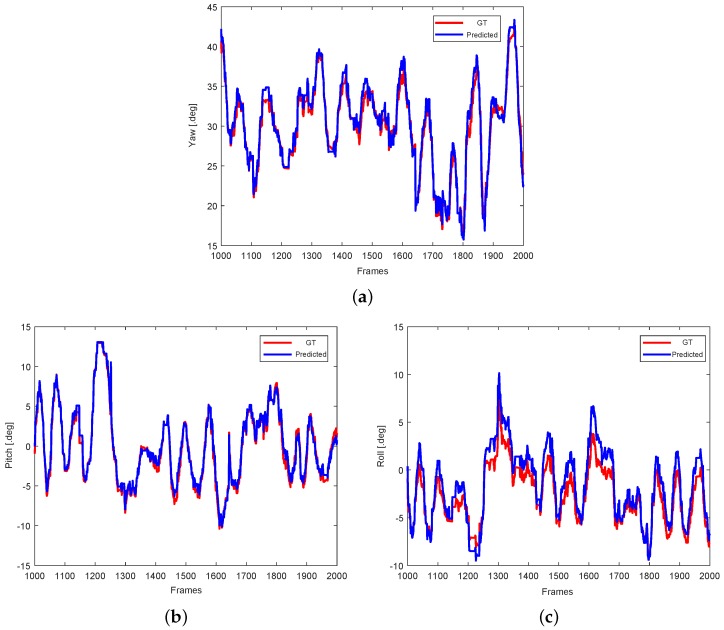
Comparisons of GT (Ground-truth) and predicted head pose value on (**a**) yaw, (**b**) pitch, and (**c**) roll.

The head pose template data for each gaze zone is generated with calibrated head pose values. We collected a standard data set of head poses for each gaze zone using a head motion sensor. Our estimated angle degree of head pose was multiplied by an expanded coefficient. The motion-sensor ground-truth data were then used to compare with the head pose computed using our algorithms. Driver head pose referring to our gaze zone are focused on areas which yaw ranges from −60 degree to +60 degree, pitch ranges from −45 degree to +45 degree and roll ranges from −10 degree to +10 degree. Figure 10 shows the tracking errors for randomly selected data segments as an example. This figure indicates that the estimation of yaw has a little large error rate than other two items, because driver rotates large angle in this dimension.

Since we apply it in a real driving environment, therefore, the tolerance error range is considered based on the gaze zone estimation. For different gaze zones, statistical results are counted (See Table 2). In this table, AME means Average Mean Error, VAR represents variance which demonstrates the derivation degree of mean error and SDR is acronyms of Success Detection Rate which is the characterization of acceptance rates in the range of tolerance. The acceptance values of yaw, pitch and roll are the estimation values whose absolute errors do not exceed the corresponding threshold (5, 2, 2 degrees for yaw, pitch and roll respectively). In Table 2, the estimation of gaze zone 1 and gaze zone 2 does not exhibit a greater stability on the edge of yaw and pitch. However, the estimations of gaze zone 6 are surrounded by more gaze zone which takes the advantages of multiple information fusion contributed by estimation of other six gaze zone. Gaze zone 3 with highest frequency is the zone when driver looks straight ahead. So, it has the highest SDR for less change of head pose.

**Figure 11:**
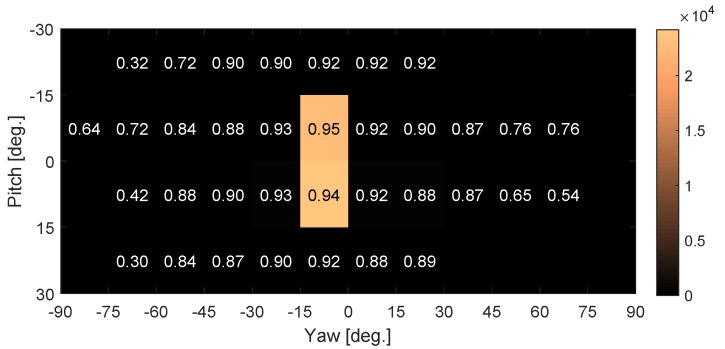
Head pose success classification rate for 5 degrees tolerance.

Figure 11 shows the visualization of accuracy rates that have been partitioned into 15×15 degrees squared regions in two-dimensional head pose (yaw and pitch). The square’s color denotes total number of frames which falls into the particular region. Since the drivers always put their attention on the road (gaze zone 3), two on-road squared regions have the most amount of frames, other squared regions have less frames. Comparing with Ref. [19], our results have a good accuracy around the zero points of head pose, and a low dynamics range of head pose, due to the multi-zone templates at particular head pose and the driving behaviour in the real-world scenarios.

When drivers turn their head from left to right in yaw, angle velocity of the head movements is really high per second. Table 3 shows the mean absolute error of head pose estimation at different head motions. All head movements that slower than 0.3 radians can be recognized as small rotation, any other measured head angle velocities are large rotation. It can be remarked that our work demonstrates the continuous head pose estimation using RGB-D sensor for natural and on-road driving. Previous works provide short-session or pre-arranged on-road evaluation [18,19]. Although the mean errors presented in these papers are less than our work’s results, it is worth noting that the evaluation performed on a head pose-free natural active driving scenario is a more practical solution to driver’s eye gaze problems. In addition, the particle filter tracking for a driver’s head state can cope with high rotation angle velocities in both yaw, pitch and roll directions.

In the evaluation, our system also has some limitations: under complex light conditions, frequent non-uniform illumination changes may result in the incomplete face region of a three-dimensional point-cloud, so the template registration processing is greatly disturbed and the head pose cannot be accurately estimated. As in Ref. [18], the sunlight sensitivity is common for vision-based systems.

### 3.3. Results of Gaze Estimation

**Figure 12:**
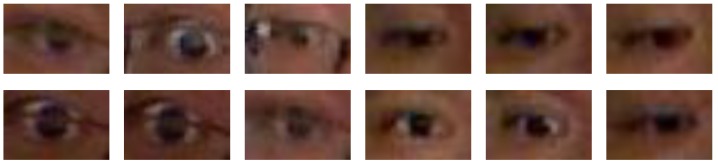
Example eye images.

While the head pose estimation is applied on the 3D point-cloud that derives from the depth data, the gaze estimation is mainly performed on the RGB data. The human eye images are cropped from the whole face images under natural illumination after facial landmark detection and anthropometry computation, and 5000 frames valid data has been retained. The cropped low-resolution human eye image are shown in Figure 12. To test the effectiveness of the proposed appearance-based gaze estimation method, some baseline appearance-based methods and model-based methods are compared on three-fold cross-validation. To be fair, all methods are compensated with the same head pose estimation (See in Section 2.1). Among them, ALR (Adaptive Linear Regression) [40] and CALR (Coupled ALR) [31] are solved using 100 selected images. The methods based on modified RF and SVR are classically trained on all training data. EGM (3D Eye Gaze model) [10] is a model-based method, and computes the gaze angle on the 3D eye model based on detected pupil center and eye center.

Table 4 depicts the results of gaze estimation with head pose compensation, and from it, it is clear that the overall gaze estimation performance for our method is better than that for other baseline methods. As expected, the appearance-based methods have certain tolerance level to non-uniform illumination, but when the light gets low, it is hard to generate discriminated features from the RGB image, due to its all values are tends to be zeros. Actually, if some eye images under dark light are exist in the training data, the performance of the appearance-based methods will be much better. However, it is much harder for model-based method to locate the pupil center or iris center, which makes the model-based method unstable under difference head pose variations and illumination variations.

### 3.4. Analysis of Gaze Zone Frequency

This part reports the experimental results of gaze zone frequency and transfer probability. Note that in a real car scenario, driver gaze zone frequency represents the state of a driver’s attention off/on the road for visual distraction, and the transfer probability provides the driver’s probable gaze zone in the next frame or motion, depicts the driver’s driving behavior habit in a temporal context. The frequencies of different driver gaze zones during driving is analyzed in Table 5. It can be seen that the gaze zone 3 (bottom left region of windshield) cases have the highest frequencies both on estimation with head pose solely, and with head pose and gaze pose. The other gaze zones with high frequency are gaze zone 2 (right mirror), gaze zone 1 (left mirror) and gaze zone 4 (rear-view mirror), which are in-car components that drivers often look at. This is consistent with the characteristics in real driving conditions. Note that the frequencies of gaze zone 1 and gaze zone 2 are almost the same. In addition, the gaze zone 9 is with the lowest frequency of gaze, because in the case of driving, it is a place that the drivers seldom glance at. In Table 5, it is clear that the gaze zone frequencies are similar between the estimation with gaze pose and without gaze pose. More specifically, the index of gaze zone with highest frequency is the same and other gaze zones’ frequencies are slightly changes. The frequency of gaze zone 3 is too large in estimation without gaze pose, when joint the gaze pose and head pose, the frequencies of its neighbor gaze zone are increased. The main reason for this is that during driving, the driver will use more eye movement while looking ahead. However, the gaze zone estimation systems with only head pose usually ignore this situation and put focus on coarse gaze directions. Therefore, the combination of head pose estimation and gaze estimation for gaze zone classification is more reasonable and accurate.

**Figure 13:**
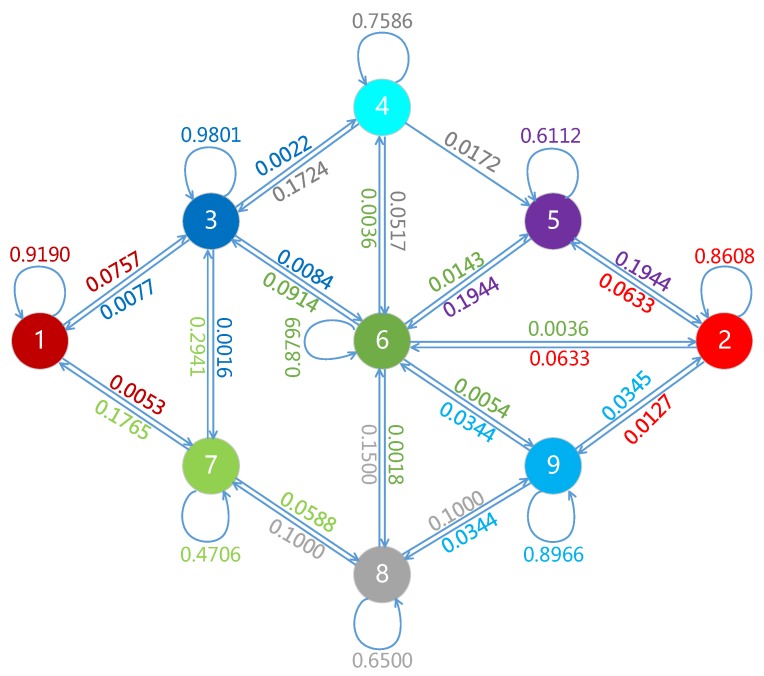
Transfer probability between different driver gaze zone.

Figure 13 provides a statistical analysis of the transfer probability between different gaze zones of 50,000 frame data from natural and on-road driving. Here, the transfer probability is defined as the frequencies of gaze zone when driver’s attention convert from current image frame to next image frame. In Figure 13, the directions that those arrows point to, are the probable gaze zone for next frame, and the numbers range in [0,1] are the corresponding probability values. For example, the probability in gaze zone 1 that remains itself is 0.9190, and that transfers to gaze zone is 0.0757, and that goes to gaze zone 7 is 0.0053. Generally, driver will look at each predefined gaze zone during a period of few seconds, resulting in many frames duration of the self transfer. Overall, most of gaze zones have high transfer probability to gaze zone 3 except themselves. This is a quick gaze return to the on-road gaze zone after viewing other gaze zones. It should also be noted that the gaze zone 4 to gaze zone 5 is a single-way transfer, that is, the driver never look at gaze zone 4 after gaze zone 5. When the driver’s gaze stays in a certain gaze zone for a long time, the driving assistance system could alarm the distraction state of the driver by the dynamic transfer probability of the gaze zone.

## 4. Conclusions

In this paper, we introduce an application-oriented solution for continuous driver gaze zone estimation systems, using multi-zone ICP-based point-cloud alignment for head pose estimation and two-stage neighbor selection for appearance-based eye gaze estimation. To accelerate the convergence speed of the ICP iteration, we utilize multi-zone templates and particle filter tracking to initialize and update the best transformation of source face template. Based on the characteristic features of head pose, and eye images, we apply a cascaded structure for a neighbor selection framework to select the nearest neighbor data that is more similar to the test head pose and eye images. Then, through the local regression of selected nearest neighbor data, a gaze estimation model is built for current gaze angle prediction. With the described solution, head orientation and gaze angle are calculated, and the gaze zone is determined by the gaze angle with head pose compensation. Our system has a reliable performance on head pose tracking and gaze estimation, making it applicable for in-vehicle driver monitoring applications. In the future, we hope to improve the accuracy of appearance-based gaze estimation in real driving environments, and to perform another study on cross-subject gaze zone estimation. 

## Figures and Tables

**Table 1:** Review of driver gaze zone Estimation Methods/Systems Using an RGB-D Camera.

Research Study	Head Pose (HP)	Gaze (GA)	Gaze Zone Estimation	Datasets Resolution
Peláez C. et al. [18]	Yaw, Pitch, Roll (ICP + Alignment )	-	HP	Continuous
Bär et al. [19]	Yaw, Pitch (Multi-template ICP + Alignment)	Yaw, Pitch (Eye Model)	HP + GA	Discrete
This study	Yaw, Pitch, Roll (Multi-zone ICP + Alignment)	Yaw, Pitch, Roll (Appearance)	HP + GA	Continuous

**Table 2:** Statistical Results for Head Pose Estimation.

Gaze Zone	Yaw			Pitch			Roll		
	AME(deg.)	VAR	SDR(%)	AME(deg.)	VAR	SDR(%)	AME(deg.)	VAR	SDR(%)
1	5.272	3.159	88.83	2.411	2.085	88.67	2.455	2.334	87.16
2	5.254	3.273	88.78	2.652	2.386	87.35	2.546	2.462	87.60
3	4.370	2.585	94.54	1.790	1.504	92.54	1.724	1.041	93.91
4	4.772	2.582	92.37	2.556	2.124	89.26	2.472	2.427	88.34
5	4.682	2.537	91.81	2.430	2.427	87.85	2.527	2.460	87.41
6	4.576	2.189	93.22	2.475	1.945	89.46	1.646	1.934	92.66
7	4.621	2.982	91.47	2.083	2.057	90.45	2.334	2.435	88.63
8	5.082	2.964	92.22	2.546	2.024	89.70	2.234	2.516	89.14
9	5.156	3.022	91.87	2.723	2.083	88.45	2.362	2.411	88.77

AME: Absolute Mean Error, VAR: Variance, SDR: Success Detection Rate of gaze zone.

**Table 3:** The Mean Absolute Error of head pose estimation at different head motions.

Head State	Yaw(deg.)	Pitch(deg.)	Roll(deg.)
Small Rotation (Proposed)	3.934	1.652	1.425
Large Rotation (Proposed)	5.797	2.562	2.848
Overall (Proposed)	4.493	1.925	1.852
Peláez C. et al. [18]	3.7	2.1	2.9

**Table 4:** The Mean Absolute Error of gaze estimation using head pose and gaze angle.

Methods	AME(deg.)
Head pose, HOG + Modified RF [41]	8.0234
Head pose, HoG + SVR [42]	8.7216
Head pose, ALR [40]	12.723
Head pose, CALR [31]	10.39
Head pose, EGM [10]	9.58
Head pose, PLSR (Proposed)	7.5682

**Table 5:** Frequency value of each gaze zone estimation.

Gaze Zone No.	Frequency only with Head Pose (%)	Frequency with both Head Pose and Gaze Angle (%)
1	5.2	4.5
2	4.9	4.7
3	81.2	74.4
4	3.5	4.4
5	2.1	4.0
6	1.6	2.8
7	0.5	1.4
8	0.9	3.1
9	0.1	0.7
